# Haplotype-resolved assembly of auto-polyploid genomes via combining Hi-C and gametic data

**DOI:** 10.1038/s41598-024-58623-5

**Published:** 2024-04-03

**Authors:** Xiaohui Zhang, Dongxi Li, Weihua Pan

**Affiliations:** 1 College of Computer Science and Technology, Taiyuan University of Technology, Taiyuan, 030024 Shanxi China; 2 Shenzhen Branch, Guangdong Laboratory for Lingnan Modern Agriculture, Genome Analysis Laboratory of the Ministry of Agriculture and Rural Affairs, Agricultural Genomics Institute at Shenzhen, Chinese Academy of Agricultural Sciences, Shenzhen, 518120 China

**Keywords:** Haplotype-resolved assembly, Auto-polyploid, PacBio HiFi, Hi-C, Gametic data, Next-generation sequencing, Haplotypes, Polyploidy, Polyploidy in plants, Genetics, Haplotypes

## Abstract

Haplotype-resolved genome assembly plays a crucial role in understanding allele-specific functions. However, obtaining haplotype-resolved assembly for auto-polyploid genomes remains challenging. Existing methods can be classified into reference-based phasing, assembly-based phasing, and gamete binning. Nevertheless, there is a lack of cost-effective and efficient methods for haplotyping auto-polyploid genomes. In this study, we propose a novel phasing algorithm called PolyGH, which combines Hi-C and gametic data. We conducted experiments on tetraploid potato cultivars and divided the method into three steps. Firstly, gametic data was utilized to bin non-collapsed contigs, followed by merging adjacent fragments of the same type within the same contig. Secondly, accurate Hi-C signals related to differential genomic regions were acquired using unique k-mers. Finally, collapsed fragments were assigned to haplotigs based on combined Hi-C and gametic signals. Comparing PolyGH with Hi-C-based and gametic data-based methods, we found that PolyGH exhibited superior performance in haplotyping auto-polyploid genomes when integrating both data types. This approach has the potential to enhance haplotype-resolved assembly for auto-polyploid genomes.

## Introduction

Haplotype-resolved genome assembly plays a crucial role in elucidating allele-specific functions. In terms of methodology, while the current assembly pipeline with PacBio HiFi based contig assembly and Hi-C based scaffolding has been able to generate phased diploid genome routinely, it is still an extremely challenging task to obtain haplotype-resolved assembly for auto-polyploid genWomes. As far as we know, there are only a very limited number of algorithms and tools existing for assembling polyploid genomes in a phasing way. These methods can be divided into three categories. The first category is reference-based phasing, which involves aligning reads to a reference genome, analyzing the variation, and using additional linkage information such as long reads or Hi-C data to phase the detected variations. For example, alignment-based haplotype phasing algorithms include minimum error correction (MEC)^1^, weighted minimum letter flip (WMLF), and maximum fragment cut (MFC)^2^. Tools like WhatsHap^3^, HapCUT2^4^ and SHAPEIT3^5^ belong to this category. This method is able to detect and phase various types of small variations, including SNPs, Indels, and small structural variations, but it usually ignores large structural variations between haplotypes. The second category is assembly-based phasing, in which haplotype-resolved contigs are directly assembled from sequencing data, followed by haplotype-resolved scaffolding with additional information such as Hi-C data for ordering the contigs and further assembling to chromosome-level. Tools such as 3D-DNA^6^, SALSA2^7^, and YaHS^8^ belong to this category. As far as we know, ALLHiC^9^ is currently one of the few specialized haplotype-resolved scaffolding tools specifically designed for auto-polyploid genomes. It is applicable to polyploid genomes with different levels of complexity, showing good performance on homologous tetraploid and octoploid sugarcane genomes^10^. However, based on our experiments, the assembly-based method is easy to generate imbalanced haplotypes (haplotypes with significantly different lengths) on contigs with a large proportion of collapsed sequences. The third category is gamete binning^11^, which performs phasing according to the similar gametic coverage profile of contigs belonging to the same haplotypes. The whole process involves single-cell DNA sequencing of hundreds of gametic genomes, aligning the short reads to contigs, building the feature vector for each contig with each component representing the read coverage of each gamete on it, and clustering by feature vectors. This method is particularly suitable for complex genomes and can address phasing imbalance issues by Sun et al.^12^. However, it has the drawback of high cost for single-cell sequencing, and accurate phasing requires a large number of gametes. For example, in Sun et al.^12^, seven hundred gametes were used for assembling a tetraploid potato genome. Overall, there is no inexpensive and effective method for haplotyping auto-polyploid genomes until now.

## Results

To validate the performance of PolyGH, we compared it with both Hi-C-based methods and gametic data-based methods separately. For the Hi-C-based approach, we chose ALLHiC. To assess the ability of the ALLHiC pipeline in separating homologous chromosomes, we assigned the contigs assembled by HiFiasm^13^ to individual chromosomes by aligning them to reference genome first, and then ran ALLHiC pipeline on each chromosome to obtain the haplotypes. As for the gametic data-based method, since the borrowed method was originally designed for potato samples, we made modifications to make it a universal approach for comparison. Table 1 presents the phasing error sizes of the three methods under varying gamete counts. Experimental observations indicate that a reduction in the chosen gamete count results in a decline in precision. Moreover, the integration of Hi-C and gametic data (PolyGH) exhibits superior performance compared to the sole utilization of gametic data (universal version of method modified from Sun et al.^12^) or Hi-C data (ALLHiC).

**Table 1:** The sizes of haplotype phasing errors for the three methods under different gamete counts.

Number of gametes	ALLHiC (bp)	Gametic data-based method (bp)	PolyGH (bp)
200	1,850,361,580	729,272,316	503,735,616
220	1,850,361,580	636,592,278	330,587,256
240	1,850,361,580	418,703,263	232,918,845
260	1,850,361,580	393,244,925	230,009,472
280	1,850,361,580	385,034,911	225,608,902
310	1,850,361,580	403,906,072	225,122,534
350	1,850,361,580	384,581,778	222,941,413
400	1,850,361,580	318,393,216	204,521,069
450	1,850,361,580	297,187,279	198,906,836
500	1,850,361,580	344,942,532	195,749,649
550	1,850,361,580	308,872,825	186,967,690
600	1,850,361,580	241,210,335	169,387,536
650	1,850,361,580	236,203,111	159,828,914
680	1,850,361,580	231,364,351	146,978,975

### Running three methods

#### Data preprocessing

We executed the pipeline specifically designed by the authors for tetraploid potato cultivars regarding the phasing of gametes (GitHub-schneebergerlab/GameteBinning).Incorporating PacBio HiFi reads downloaded earlier, short reads from single-cell sequencing, and Illumina sequencing data from 10× single-molecule libraries, we completed steps 1 to 10 of the pipeline to perform in-depth analysis of contigs.

#### Combing Hi-C and gametic data

PolyGH first utilizes the Jellyfish count command for k-mer counting. The parameters are set to “-m 21 -s 3G -c 7,” resulting in a binary file containing k-mer count information. This file records each k-mer of length 21 that appears exactly once in the input sequence. If multiple hash files are generated, they need to be merged using the merge command. Since the count command produces binary results, the dump command with parameters “-c -t -U 1” is used to export and convert the k-mer count results into a human-readable text file. Each line represents a k-mer and its corresponding frequency. Next, the rafilter tool is employed for k-mer localization and querying. To begin, the rafilter build subcommand constructs a k-mer position library. This library file records the positions of k-mers within the input sequence, facilitating subsequent data analysis and processing. Given that Hi-C data consists of paired-end reads, it is essential to obtain information about k-mer positions on contig fragments and the positions of contig-based k-mers within Hi-C paired-end reads. Finally, the PolyGH pipeline is executed to extract Hi-C signals and cluster contigs. The results include Hi-C signal files between contig fragments and contig grouping information related to Hi-C and gametic data. As a result, contigs can be assigned to four different haplotypes, each comprising 12 chromosomes. We employed PolyGH to assess assembly quality.

Taking the tetraploid potato cultivar test data as an example, the authors achieved a successful assembly using 717 selected nuclei (3.1 Gb haplotype-resolved at 99.6% precision). From this dataset, we randomly selected 200, 220, 240, 260, 280, 300, 350, 400, 450, 500, 550, 600, 650, and 680 nuclei for gamete binning experiments. Through experimentation, it was observed that reducing the number of gametes used led to a decrease in accuracy.

#### Using gametic data

Since running the ‘gamete_binning_tetra’ script directly would result in errors for low gametes, and since the process for handling haplotigs is the same, we modified the code provided by Sun et al.^12^ to implement the direct application of its process for haplotig-type contig fragments. For collapse fragments, we utilized the constructed gametic feature vectors and allocated diplotig-type fragments to the two groups within the same homologous chromosome set that had the highest similarity based on the feature vectors. Similarly, triplotig and tetraplotig fragments can be associated with three and four groups respectively.

#### Using Hi-C data

Taking tetraploid potato cultivar samples as an example, we run the ALLHiC pipeline with parameters set to “-k 48 -e MBOI”. This resulted in a final fasta file (groups.asm.fasta) containing 48 chromosomes and unidentified scaffolds. To attain enhanced outcomes and validate the capability of the ALLHiC pipeline to distinguish homologous chromosomes, we initially aligned the preliminarily assembled contig sequences with the recently assembled DM genome using minimap2 with parameters “-cx asm5 --eqx”. If the alignment length of a contig sequence with a reference chromosome was the longest, we considered the contig to belong to that chromosome. Consequently, the contigs were assigned to the 12 chromosomes. Running the ALLHiC pipeline on individual chromosomes with the parameters “-k 4 -e GATC”, we obtained four haplotypes for each of the 12 chromosomes. Subsequently, we evaluated the assembly outcomes of ALLHiC.

### Results evalution

Utilizing a tetraploid potato cultivar dataset, we achieved a robust assembly of the *S*. *tuberosum* ‘Otava’ genome through single-cell sequencing of 717 pollen genomes, resulting in a high-quality 3.1 Gb haplotype-resolved assembly with 99.6% precision. This reference assembly was then employed to evaluate three distinct methods. For contig fragments exhibiting discordant grouping, we quantified size errors using the formula: size_error = size_haplotig * 1 + size_diplotig * n_diplotig + size_triplotig * n_triplotig + size_tetraplotig * n_tetraplotig. In this context, size_error refers to the overall error size, while size_haplotig, size_diplotig, size_triplotig, and size_tetraplotig indicate the sizes of haplotig, diplotig, triplotig, and tetraplotig-type contig fragments, respectively. Furthermore, n_diplotig, n_triplotig, and n_tetraplotig represent the count of contig fragments of diplotig, triplotig, and tetraplotig types deviating from the reference grouping. ALLHiC relies on the initial contig assembly quality as it extracts phased assembly information from Hi-C sequencing data. Excessive chimeric and collapse sequences can lead to an error size of around 1.85 Gb in ALLHiC results. Conversely, our approaches, focusing on gametes and integrating gametes with Hi-C data, were tested using various pollen genomes, as indicated in the table above. This fusion of data types substantially minimizes contig phasing errors and yields more precise outcomes.

## Discussion

In this study, we proposed a novel phasing algorithm called PolyGH for haplotyping auto-polyploid genomes. Currently, there are limited methods available for haplotyping auto-polyploid genomes, and our research aimed to address this challenge. The existing methods for haplotype-resolved assembly can be categorized into three groups, and all contain some flaws. Reference-based phasing methods often ignore large structural variations between haplotypes. Assembly-based phasing may lead to imbalanced haplotypes and collapsed sequences. Gamete binning, on the other hand, requires single-cell DNA sequencing and is suitable for complex genomes, but it is costly and requires a large number of gametes. Our experiments were conducted on tetraploid potato cultivars using the PolyGH algorithm. By integrating Hi-C and gametic data, PolyGH showed promising results in haplotype-resolved assembly for auto-polyploid genomes. However, further validation and testing on other polyploid genomes are needed to assess its performance and generalizability.

In conclusion, our proposed PolyGH algorithm addresses the challenges of haplotype-resolved assembly for auto-polyploid genomes. By combining Hi-C and gametic data, we improve the accuracy of phasing and provide a potential solution for inexpensive and effective haplotyping of auto-polyploid genomes. Since currently only tetraploid potato cultivars are available for the data integration, future research can explore the application of PolyGH on other polyploid genomes and optimize its performance for large-scale assembly projects.

## Materials and methods

We obtain contig sequence of tetraploid potato cultivar Otava from the study by Sun et al.^12^. Since currently only tetraploid potato cultivars are available for the data integration, we conducted our experiments on this dataset. In this paper, we propose a novel phasing algorithm called PolyGH by combing Hi-C and gametic data. The method can be divided into three steps.

In the first step, PolyGH bins the non-collapsed contigs using only gametic data. First, the contigs are cut into fragments of 10 kb. Second, the single-cell short reads of each gamete are aligned^14,15^ to the fragments, and the coverage of each fragment is calculated through samtools^16^. Third, the fragments are classified into four types: haplotigs, diplotigs, triplotigs, and tetraplotigs according to the coverages. Fourth, the adjacent fragments of the same type within the same contig were merged into larger fragments of 50 kb. When constructing feature vectors, we initially gathered read counts for contig fragments that aligned to varying numbers of selected single cells. Simultaneously, we computed the average read count for each individual cell. Using this information, we determined the coverage of contig fragments within the selected single cells. Subsequently, we constructed feature vectors based on distinct single-read sets. Finally, leveraging the gametic feature vectors, we clustered haplotigs based on the similarity between these vectors. Since the first step of PolyGH is similar to the corresponding process in Sun et al.^12^, we have re-implemented the code and optimized the method. During the experimental process, we performed data preprocessing before alignment. This included removing low-quality reads and PCR duplicates. After alignment, we conducted post-processing and filtering on the data. For instance, we removed low-coverage fragments, eliminated duplicate alignments, and filtered out low-quality alignments.

The second step is to obtain accurate Hi-C signals. Through experiments, it was found that reducing the number of selected single cells would reduce accuracy. It was observed that this module performs well for non-collapsed contig clustering, and the main factor affecting accuracy is collapsed segments. Therefore, we comprehensively utilize gamete and Hi-C information to cluster collapsed segments. First, we need to obtain accurate Hi-C signals. Due to the high similarity between the homologous sequences on different haplotypes, there may be also strong Hi-C signals between contigs from different haplotypes misleading the phasing process. The conventional method for capturing Hi-C signals involves aligning Hi-C paired-end reads to contigs. However, this approach has its limitations. For instance, the presence of repetitive regions in the genome can make it challenging to accurately distinguish interactions within these regions through direct alignment. Furthermore, longer contigs may combine interactions from multiple chromosomes.

To solve this problem, in PolyGH, we only use the Hi-C signals related to differential genomic regions such as SNPs and structural variations. To consistently process these variations, we use unique k-mer (the genomic subsequence of length k appearing in all contigs for only once) to represent them. More specifically, Jellyfish is used to count the appearance of each specific k-mer, and the ones with count one are selected as unique k-mers. Then the positions of these unique k-mers on contigs are recognized by a module in our previous algorithm RAfilter^17^. To extract Hi-C signals between contigs, the initial task is to determine the contig assignment for Hi-C paired-end reads. This is accomplished by performing an intersection operation between the unique k-mers of the reads and the contig sequences. By identifying the contig with the largest intersection, indicating a higher number of shared unique k-mers, we assign the read to that particular contig. If the Hi-C paired-end reads belong to different contigs, we consider it as evidence of Hi-C signals between those contigs, with the signal strength progressively increasing. Conversely, if the Hi-C paired-end reads belong to the same contig, the signal is disregarded. The raw Hi-C signals between contigs are normalized by the product of the numbers of unique k-mers on them. Using unique k-mers to determine Hi-C signals between two fragments is an approximate method, and the signal results may be affected by various factors such as the length of fragments. Therefore, we can integrate gamete information to determine chromosomal grouping.

In the third step, PolyGH assigned the collapsed fragments (diplotigs, triplotigs, and tetraplotigs) into the groups of haplotigs according to the combined Hi-C and gametic signals. The gametic signals are obtained by calculating the similarities between gametic feature vectors. Based on the above grouping of haplotigs, the signals of the collapsed fragment to each haploid group are calculated. For gamete signals, the strongest signal among all signals from this collapsed fragment to the haplotigs of one group is counted as the signal from this fragment to this group. The correlation of each fragment with the haploid group is calculated individually and normalized to 0–1. For Hi-C signals, the Hi-C signals between the fragment and haplotigs in each haploid group are calculated separately first, and then normalized: ${\text{signal}}\times {10}^{8}/sum\left({\text{haplotig}}\_{\text{size}}\right)$, where the normalization factor is the sum of sizes of haplotigs related to the collapsed fragment in each group. This result is taken as the signal from this fragment to each haploid group, and the results are normalized to 0–1. To combine these two different types of signals, PolyGH calculates the weighted sum of them and the weights represent their importance accordingly. Due to the difficulty of deciding the weights, PolyGH uses an iterative algorithm that deals with fragments from larger to smaller progressively and tunes the weight of Hi-C signals higher and higher, adjusting the weights of Hi-C signals from 10 to 50%. The reason for this operation is that the Hi-C signals are in comparatively higher resolution than gametic ones, and thus are more effective for solving shorter fragments.

**Figure 1:**
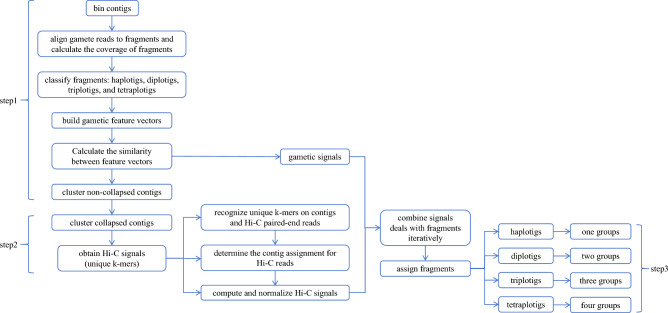
Algorithm flowchart; the input to the algorithm is contig sequence of polyploid genomes.

For each ungrouped fragment, the combined signal to each haplotig is calculated. Each haplotig, diplotig, triplotig, or tetraplotig is assigned to one, two, three, or four groups respectively with the strongest signals from it. If the fragment type is haplotig, the fragment is assigned to the haploid group with the strongest signal as its final group; while assigning collapsed fragments, first find the homolog cluster containing the haploid group with the strongest signal to the fragment. For diplotig types, the fragment will be assigned to the two groups within the homolog cluster with the strongest signals. For triplotig types, the fragment will be assigned to the three groups within the homolog cluster with the strongest signals, excluding the haploid group with the strongest signal. When dealing with tetraplotig types, relying solely on individual gametic data can lead to information gaps. For contig fragments composed entirely of tetraplotigs, the absence of linking information makes contig grouping impossible. However, PolyGH offers a solution by leveraging Hi-C signals to group contig fragments effectively. The pipeline of the algorithm is illustrated in Fig. 1.

## Data Availability

The Hi-C sequencing, PacBio HiFi sequencing, Illumina sequencing of 10× single-molecule libraries and Single cell sequencing of Potato cultivar Otava pollen are downloaded from SRA Links for BioProject (Select 726019)—SRA—NCBI (nih.gov), The data was downloaded in its original format through Amazon Simple Storage Service. The contig sequence of the potato cultivar Otava can be downloaded from the following link: https://mega.nz/folder/GktXEYCR#F3I8uTKvKO0Fu8VY2yc2WA. The genome assembly and Genome information for DM used in this study are downloaded from Spud DB (Spud DB (uga.edu)).

## Code availability

Custom code and scripts supporting this work are available at https://github.com/zhangxiaohui999/PolyGH.
